# Data for accelerated degradation of calcium phosphate surface-coated polycaprolactone and polycaprolactone/bioactive glass composite scaffolds

**DOI:** 10.1016/j.dib.2016.01.023

**Published:** 2016-01-22

**Authors:** Patrina S.P. Poh, Dietmar W. Hutmacher, Boris M. Holzapfel, Anu K. Solanki, Maria A. Woodruff

**Affiliations:** a Institute of Health and Biomedical Innovation, Queensland University of Technology, 60 Musk Avenue, Kelvin Grove, 4059 Brisbane, Australia; b Department of Experimental Trauma Surgery, Klinikum Rechts der Isar, Technical University Munich, Ismaninger 22, 81675 Munich, Germany; c Institute for Advanced Study, Technical University Munich, Lichtenbergstrasse 2a, 85748 Garching, Munich, Germany; d Department of Orthopaedic Surgery, Koenig-Ludwig Haus, University of Wuerzburg, Brettreichstr. 11, 97074 Wuerzburg, Germany; e Department of Materials and Department of Bioengineering, Institute of Biomedical Engineering, Imperial College London, Exhibition Road, London SW7 2AZ, United Kingdom

## Abstract

Polycaprolactone (PCL)-based composite scaffolds containing 50 wt% of 45S5 bioactive glass (45S5) or strontium-substituted bioactive glass (SrBG) particles were fabricated into scaffolds using melt-extrusion based additive manufacturing technique. Additionally, the PCL scaffolds were surface coated with a layer of calcium phosphate (CaP). For a comparison of the scaffold degradation, the scaffolds were then subjected to *in vitro* accelerated degradation by immersion in 5 M sodium hydroxide (NaOH) solution for up to 7 days. The scaffold׳s morphology was observed by means of SEM imaging and scaffold mass loss was recorded over the experimental period.

## **Specifications table**

**Table:** 

Subject area	*Tissue engineering*
More specific subject area	*Biomaterials*
Type of data	*SEM images and graph*
How data was acquired	*SEM (*FEI Quanta SEM/FIB) *Electronic balance with 0.1 mg resolution*
Data format	*Analyzed*
Experimental factors	*Composite made of polycaprolactone (PCL) and 50 wt% of bioactive glass (45S5 bioactive glass (45S5) or strontium-substituted bioactive glass (SrBG)) were synthesized by fast precipitation into excess ethanol. PCL and composite (PCL/50-45S5 and PCL/50-SrBG) scaffolds were fabricated by means of additive manufacturing. Additionally, PCL scaffolds were coated with a layer of calcium phosphate.*
Experimental features	*PCL, PCL-CaP coated, PCL/50-45S5 and PCL/50-SrBG scaffolds were subjected to an accelerated in vitro degradation study using 5* *M NaOH solution.*
Data source location	*Brisbane, Australia*
Data accessibility	*Data in article*

## **Value of the data**

• The data can be used as a benchmark to study and compare the degradation behaviors of new polymers and polymeric scaffolds.
• By directly comparing the stability of both bioactive glass and calcium phosphate, our approach provides new insights into the stability of surface coating vs embedded particles.
• The data shows a direct comparison of the degradation behavior of PCL, PCL-CaP coated, PCL/50-45S5 and PCL/50-SrBG scaffolds under an accelerated rate.

## 1 Data

**Fig. 1:**
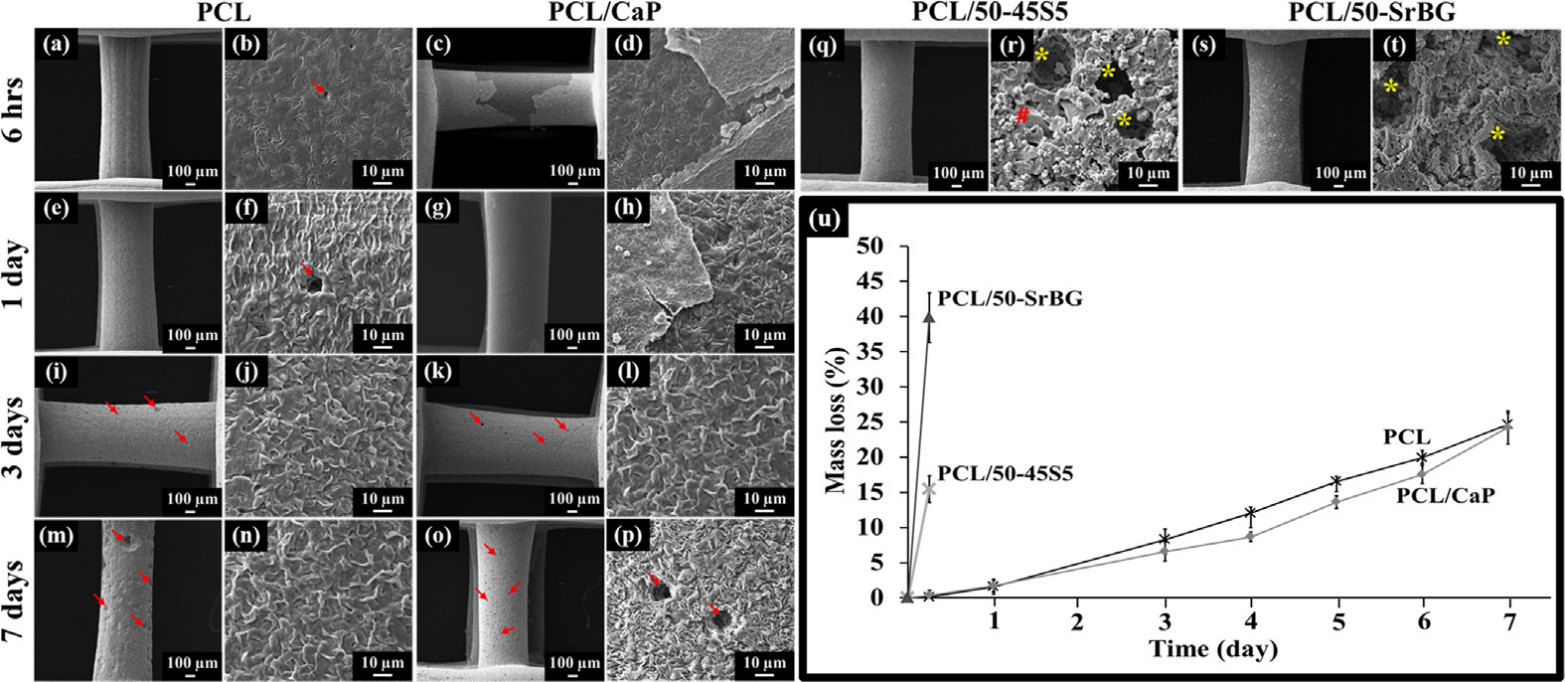
(a–p) SEM images of PCL and PCL/CaP scaffolds after 6 h, 1, 3 and 7 days immersion in 5 M NaOH. (q–t) SEM images of PCL/50-45S5 and PCL/50-SrBG scaffolds after 6 h immersion in 5 M NaOH. After 24 h, all PCL/50-45S5 and PCL/50-SrBG scaffolds were disintegrated into pieces and not retrievable for further analysis. Red arrows (→) show presence of pits on PCL or PCL/CaP scaffold surfaces. Yellow * represents pits on PCL strut due to dislodgement of BG particles. Red # indicates BG particles. (u) Percentage mass loss of PCL, PCL/CaP, PCL/50-45S5 and PCL/50-SrBG scaffolds over time when immersed in 5 M NaOH, at 37 °C. *n*=8, Mean±SD. (For interpretation of the references to color in this figure legend, the reader is referred to the web version of this article.)

The *in vitro* degradation of PCL, PCL/CaP, PCL/50-45S5 and PCL/50-SrBG composite were evaluated using an *in vitro* accelerated degradation test as previously reported by Lam et al. [1]. Over 7 days of immersion in 5 M NaOH, PCL and PCL/CaP scaffolds lost 24.6±1.6% and 24.2±2.4% of their original mass respectively (Fig. 1u). Through SEM observation of the surfaces of scaffolds’ struts over time, it was revealed that both PCL and PCL/CaP scaffold surfaces were roughened and that an increasing numbers of pits were observed on the surfaces (Fig. 1). After 6 h immersion in 5 M NaOH, it was observed that the CaP coating on PCL/CaP scaffolds detached from the scaffold surfaces, and it was completely absent from the scaffold surfaces after 3 days (Fig. 1). On the other hand, with only 6 h immersion in 5 M NaOH, PCL/50-45S5 lost 15.5±1.9% of its original mass and PCL/50-SrBG lost 39.84±3.56% of its original mass (Fig. 1u). After 24 h immersion, both PCL/50-45S5 and PCL/50-SrBG scaffolds had disintegrated into pieces and were not retrievable for further analysis. Although the struts of PCL/50-45S5 and PCL/50-SrBG scaffolds appeared intact after 6 hours immersion in 5 M NaOH (Fig. 1q and s), inspection by SEM of the scaffold surfaces showed numerous large pits, which was due to dislodging of the BG particles from the PCL microfilaments (Fig. 1r and t). The surfaces were also much rougher compared to PCL and PCL/CaP scaffolds immersed in 5 M NaOH for the same period of time.

## 2 Experimental design, materials and methods

### 2.1 Composite material synthesis and scaffold fabrication

50 wt% of 45S5 and SrBG particles (≤20 µm) with the composition of 46.13 SiO_2_ – 2.60 P_2_O_5_ – 24.35 Na_2_O – 26.91 CaO (mol%) and 46.13 SiO_2_ – 2.60 P_2_O_5_ – 24.35 Na_2_O – 6.73 CaO – 20.18 SrO) (mol%) [2], respectively, were incorporated into the PCL (CAPA 6500, Perstorp, United Kingdom) bulk by fast precipitation into excess ethanol [3]. Detailed description of the composite synthesizing procedures can be found in Poh et al. [4].

Once the composite materials were air-dried with a constant weight over 3 consecutive days, scaffolds (PCL, PCL/50-45S5, and PCL/50-SrBG) were fabricated by mean of additive manufacturing technology at 90 °C. All scaffolds with the dimension of 50 (*L*)×50 (*W*)×2.4 (*H*) mm^3^ were fabricated using a 21 G nozzle, with a lay-down pattern of 0–90°, filament gap of 2 mm and layer thickness of 0.4 mm.

### 2.2 Calcium phosphate coating on PCL scaffolds

By adapting a method described by Vaquette et al. [5], PCL scaffolds were coated with CaP. Briefly, PCL scaffolds were immersed in 70% ethanol under vacuum for 10 min to remove entrapped air bubbles. Then, scaffolds were placed in pre-warmed 5 M NaOH solution (37 °C) under vacuum for 10 minutes followed by incubation at 37 °C for 60 min using a water bath. The scaffolds were rinsed with MilliQ water until the pH of the rinsing water was ~pH 7. Then, the scaffolds were immersed in filtered 10× simulated body fluid (SBF) adjusted to pH 6 with sodium bicarbonate (NaHCO_3_) (initially described by Kokubo et al. [6]) at 37 °C for 60 min with one change of fresh filtered 10× SBF solution after 30 min. Then, the scaffolds were rinsed twice with MilliQ water, and immersed in 0.5 M NaOH at 37 °C for 30 min to homogenize the coated CaP phase. Finally, the scaffolds were rinsed with MilliQ water until the rinsing solution reached ~pH 7.

### 2.3 Accelerated degradation of scaffolds in 5 M NaOH

Scaffolds of 50 (*L*)×50 (*W*)×2.4 (*H*) mm^3^ were cut into 4×4×2.4 mm^3^ and immersed in 5 M NaOH (Sigma-Aldrich) and maintained at 37 °C for 6 h, 1 day, 3, 4, 5, 6, and 7 days to recapitulate the initial degradation of PCL in vitro*,* but at an accelerated rate as described by Lam et al. [1]. The initial and final mass (dried under vacuum for 48 h) of each scaffold was measured using an electronic balance with 0.1 mg sensitivity and the percentage of mass loss was calculated. Scaffold morphology was examined using SEM operating at 10 kV after gold sputter-coating.
